# Roles of core *nosZ* denitrifiers in enhancing denitrification activity under long-term rice straw retention

**DOI:** 10.3389/fpls.2025.1541202

**Published:** 2025-02-07

**Authors:** Shijie Zhang, Mengyao Hou, Bing Li, Panfeng Guan, Qing Chi, Hao Sun, Hangbo Xu, Dongjie Cui, Yupan Zhu

**Affiliations:** ^1^ Zhengzhou Research Base, State Key Laboratory of Cotton Bio-breeding and Integrated Utilization, School of Agricultural Sciences, Zhengzhou University, Zhengzhou, China; ^2^ Henan Key Laboratory of Ion-Beam Green Agriculture Bioengineering, Zhengzhou University, Zhengzhou, China

**Keywords:** straw retention, denitrification activity, *nosZ* gene, denitrifiers, paddy field

## Abstract

The denitrification process is known to contribute to soil nitrogen (N) loss, which is strongly affected by fertilization strategies; however, the effects of distinct straw retention modes on soil denitrification activity have rarely been discriminated and the underlying mechanisms remain unclear. This study coupled field and incubation experiments to explore the characteristics of soil denitrification activity, soil and standing water physicochemical properties, and the abundance, community diversity, and co-occurrence network of *nosZ* denitrifiers, based on a paddy field implementing 10-year straw retention under a rice–wheat rotation system. Four straw retention treatments with equivalent chemical fertilizers were applied, namely no straw (NS), wheat straw only (WS), rice straw only (RS), and wheat and rice straw (WRS). Results indicated a significant increase (by 41.93–45.80% when compared to that with NS) in the soil denitrification activity with RS and WRS. Correspondingly, treatments with rice straw retention resulted in the development of a similar community composition (*P* < 0.05), structure (*P* = 0.001), and more positively interconnected network, as well as similar specific keystone taxa of *nosZ* denitrifiers, relative to those in non-rice straw mode. Under long-term rice straw retention conditions, the core *nosZ*-denitrifying phylogroups shifted (r = 0.83, *P* < 0.001), with the recruitment of keystone taxa from the phyla Bacteroidetes and Euryarchaeota playing a key role in enhancing denitrification activity and stimulating N loss. Accordingly, in a rice–wheat rotation field, the practice of wheat straw retention in a single season is recommended because it will not markedly sacrifice soil N availability impaired by the denitrification process.

## 1 Introduction

Owing to nutrient-rich and aerobic–anaerobic interfaces (Du et al., 2024; Nie et al., 2019), the flooded paddy field serves as a hotspot for denitrification processes, which reduce nitrate (NO_3_^−^) to atmospheric nitric oxide (NO), nitrous oxide (N_2_O), or dinitrogen (N_2_) in a stepwise manner (Seitzinger et al., 2006). The end-products of heterotrophic denitrification under anoxic or microaerophilic conditions are dominated by N_2_, followed by N_2_O and NO (Jahangir et al., 2012), jeopardizing N use efficiency in agricultural ecosystems and contributing to global warming (Philippot et al., 2007). Approximately 36% of N fertilizer was estimated escape from paddy fields through denitrification pathways (Aulakh et al., 2001; Ju et al., 2009).

The practice of straw retention has long been recommended to improve soil N availability, as well as organic carbon (C) sequestration (Thangarajan et al., 2013; Xu et al., 2024); moreover, organic C sources comprise one of the crucial drivers of the denitrification process by providing it with energy and electron donors (Wang et al., 2023). However, the effects of crop residue retention on denitrification activity have varied considerably, with 2.62% to 460.0% of denitrification activity enhanced by straw amendment (Wang et al., 2021, 2022, 2024), among previous studies. Those differences among distinct straw retention modes have rarely been categorized, limiting our knowledge of how soil denitrification pathways are affected by crop residues. It warrants a thorough investigation of denitrification activity by incorporating different modes of straw retention in a two-crop rotation field, especially under the same fertilization conditions, which is critical to explore and guide field management strategies with respect to crop residues.

Microorganisms are the key factors driving soil nitrogen (N) transformation. The nitrous oxide reductase, encoded by the *nosZ* gene, is increasingly assessed to explore the abundance and structure of denitrifiers and determine the denitrification activity in the soil environment (Duan et al., 2018; Qin et al., 2021; Tao et al., 2022; Tang et al., 2024). Recent studies have highlighted the significant diversity of nosZ-denitrifying communities, which vary under different agricultural management practices, including straw retention (Chen et al., 2025; Xie et al., 2024; Zhou et al., 2022). Furthermore, it has been shown that the *nosZ* gene exists in two distinct clades (*nosZ*-I and *nosZ*-II), each contributing differently to the denitrification process under varying soil conditions (Bano et al., 2024). Despite extensive efforts regarding fertilization regimes, much less is available about the effects of different straw retention modes on *nosZ*-denitrifiers, which directly regulate the organic C sources that might regulate the ability of denitrifiers to compete for soil nitrate (NO_3_^−^) or nitrite (N_2_O) (Giles et al., 2012; Taylor and Townsend, 2010). Moreover, accumulating studies have reported insignificant relationships between denitrification activity and the abundance or composition of denitrifiers (Attard et al., 2011; Kou et al., 2019), suggesting the necessity of exploring key underlying taxa of the denitrifiers, which dominate the specific ecosystem processes in different habitats (Liang et al., 2021; Kong et al., 2023). Therefore, we hypothesized that specific keystone taxa of denitrifiers are likely stimulated under certain straw retention modes, thereby increasing the soil denitrification activity. This hypothesis was verified based on a field experiment employed with a 10-year program of straw retention. The objectives of this study were (1) to discriminate the soil denitrification activity responses across different straw retention modes, (2) to characterize the abundance, diversity, and co-occurrence network of denitrifiers, and (3) to identify the key soil and standing water factors that drive denitrification activity.

## 2 Materials and methods

### 2.1 Field experiment and sampling

The field experiment was initiated in 2010 under a summer rice (*Oryza sativa* L.)–winter wheat (*Triticum aestivum* L.) rotation at the Agro–Ecological Station of the Chinese Ecosystem Research Network in the Taihu Lake region of China. The experimental soil belongs to the category of Anthrosols and originates from lacustrine sediments. The field experiment adopted a randomized complete block design with three replicates of four treatments as follows: no straw retention (NS), wheat straw retention in the summer season (WS), rice straw retention in the winter season (RS), and wheat and rice straw return in the summer and winter seasons, respectively (WRS). All four treatment groups received the same amount of mineral N, P, and K fertilizers (**Supplementary Table S1**). Crop residues in the straw retention treatments, including rice and wheat residues, were chopped into pieces measuring 5 to 10 cm in length and incorporated into the soil in a rotary manner approximately one week before the next crop season.

During the rice season in July 2020, three replicate soil (0–15 cm) and standing water samples were randomly collected from each plot and stored in sterile plastic bags and bottles, respectively. The water samples were filtered through 0.45 μm membranes and processed for chemical analysis. The soil samples were homogenized by passing them through a 2 mm sieve, divided into three parts, and then processed for chemical analysis (stored at 4°C), soil incubation experiments (stored at 4°C), and molecular assays (stored at −80°C).

### 2.2 Determination of chemical properties

The ammonium nitrogen and nitrite nitrogen in the filtered standing water samples were measured using an ultraviolet spectrophotometer (UV-1280, Shimadzu, Japan) according to Committee of Analytical Method of Water and Wastewater (2002). The concentration of water organic nitrogen was analyzed using a Multi N/C 2100S analyzer (Analytikjena GmbH, Germany) (Ghani et al., 2003). Soil pH was measured using a portable meter (Mettler Toledo, Switzerland) at a soil:water ratio of 1:2.5. Soil electrical conductivity (EC) was detected based on a 1:2.5 soil-to-water ratio using a conductivity salinity meter (Y SI-30, Yellow Springs, USA). Soil organic carbon (SOC) and dissolved organic C (DOC) were measured using TOC-VCPH equipment (Shimadzu, Japan) based on Lu (2000). Soil NH_4_^+^-N, NO_3_^−^-N, and nitrite nitrogen (NO_2_^−^-N) were desorbed with 2 mol L^−1^ KCl (1:5 soil:solution) and analyzed with an Auto-Analyzer (Skalar, The Netherlands). Soil total nitrogen was measured using the dry combustion method (Lu, 2000). Soil organic nitrogen (SON) was calculated as the difference between soil total nitrogen and the combined soil NH_4_^+^, NO_3_^−^, and NO_2_^−^. Soil available potassium (AK) and phosphorus (AP) were desorbed with 1 mol L^−1^ NaHCO_3_ and measured by atomic absorption spectrophotometry and the molybdenum-blue method, respectively. The physicochemical properties of soil and standing water are presented in **Table 1**.

**Table 1:** Physicochemical properties of soil and standing water.

	pH	NH_4_^+^	NO_3_^−^	NO_2_^−^	SON	SOC	DOC	w-NH_4_^+^	w-NO_3_^−^	w-DON	w-DOC
		mg kg^−1^	mg kg^−1^	mg kg^−1^	g kg^−1^	g kg^−1^	mg kg^−1^	g mL^−1^	g mL^−1^	g mL^−1^	g mL^−1^
NS	7.10a	6.79c	9.05a	1.29a	2.01c	19.58c	99.2b	5.17a	2.07a	1.75b	12.66b
WS	6.95ab	9.04b	6.16c	1.00ab	2.15b	23.05b	110.5b	3.39b	1.63cd	2.09ab	14.07ab
RS	6.80b	10.23a	6.53b	0.99b	2.25ab	25.44ab	129.4a	3.73ab	1.70bc	2.18a	15.87a
WRS	6.63b	10.44a	6.44bc	1.10ab	2.38a	26.13a	135.6a	3.47b	1.79b	2.11a	16.25a

Indicators with and without “w” represent those properties in standing water and soil, respectively. Different lowercase letters in the same column indicate significant differences (*P* < 0.05) according to the Tukey’s HSD *post-hoc* test. SON, soil organic nitrogen; DOC, dissolved organic C; DON, organic nitrogen; NS, no straw; RS, rice straw only; WS, wheat straw only; WRS, rice straw and wheat straw.

### 2.3 Determination of denitrification enzyme activities

The denitrification enzyme activity (DEA) was measured in accordance with the acetylene (C_2_H_2_) inhibition method modified from Barton et al. (2000). Briefly, three replicates (10 g) of each fresh soil sample were added to 120 mL glass flasks. Each was amended with 20 mL of solution containing glucose (300 μg C^−1^ soil) and potassium nitrate (50 μg N g^−1^ soil). Each flask was sealed with rubber septa and an aluminum crimp cap, and the head space was evacuated and purged with helium gas for 1 min thereafter. Then, 10% of the headspace was replaced with C_2_H_2_ (12 mL) through injection. Finally, the replicates were incubated in the dark on a shaker (180 rpm, 25 °C) for 30, 60, 90, and 120 min. Approximately 5 mL of gas samples was transferred into pre-evacuated glass vials and determined using a gas chromatograph (GC-8A, Shimadzu, Japan).

### 2.4 DNA extraction, real-time PCR assay, and high-throughput sequencing

Genomic DNA was extracted from the triplicate subsamples (0.33 g fresh soil) with Power Soil DNA Isolation Kits (QIAGEN, USA). The quantity and quality of the extracted DNA were measured with a NanoDrop spectrophotometer (Thermo Fisher Scientific, Wilmington, USA). The extracted DNA was divided into two parts, with one fraction for real-time PCR assays and the other fraction for high-throughput sequencing.

The denitrification-associated *nosZ* gene was amplified using the primer pairs nosZF (5′-CGCRACGGCAASAAGGTSMSSGT-3′) and nosZ (5′-CAKRTGCAKSGCRTGGCAGAA-3′). The reaction mixtures (10 μL) consisted of 5 μL of 2× SYBR green mix II (TaKaRa Biotechnology Co. Ltd., Dalian, China), 0.2 μL of 50× Rox Reference Dye (TaKaRa Biotechnology Co. Ltd., Dalian, China), 0.2 μL (10 μM) of forward and reverse primers, 5 ng of DNA template, and deionized water. The amplification systems consisted of 5 μL of SYBR Master Mix, 0.2 μL (10 μM) of forward and reverse primers, 1 μL of DNA template, and 3.6 μL of double-distilled water (ddH_2_O). qPCR assay was performed at 95°C for 5 min (denaturation), followed by 40 cycles at 95°C for 30 s, 60°C for 30 s, and 72°C for 60 s (Henry et al., 2006). The qPCR amplification efficiencies and R^2^ value of the triplicates were 91.4%–95.6% and > 0.99, respectively.

High-throughput sequencing of the *nosZ* gene was performed using the same primers (nosZF/nosZR) as those for qPCR. The conditions of PCR amplification were as follows: denaturation at 95°C for 3 min; followed by 35 cycles at 94°C for 30 s, 52°C for 45 s, and 72°C for 20 s; and a final elongation at 72°C for 5 min (Delorme et al., 2003). The PCR products were then purified, quantified, and sent for paired-end sequencing (2 × 300 bp) on an Illumina MiSeq platform (Illumina, San Diego, CA, USA).

### 2.5 Statistical analysis

Raw sequence data were assembled using FLASH2 (Magoč and Salzberg, 2011) and quality filtered using VSEARCH v2.15 (Rognes et al., 2016) and unoise3 in usearch (Edgar, 2016). In total, 769136 high-quality sequences were obtained from 16 samples. The high-quality sequences were clustered into operational taxonomic units (OTUs) based on a 97% nucleotide similarity cutoff (Edgar, 2010), and 2691 OTUs were finally generated. The taxonomic annotations of the OTUs were in accordance with the RDP Functional Gene Repository (FunGene, http://fungene.cme.msu.edu/) using a confidence threshold of 80%. The sequence data have been deposited in the NCBI Sequence Read Archive under the accession number PRJNA668401.

All data were tested for homogeneity of variance with Levene’s tests before analysis. One-way analysis of variance (ANOVA), permutation analysis of variance (PERMANOVA) tests, and the Kruskal-Wallis H-test were performed with SPSS 22.0, R 3.2.1 (http://www.rproject.org/), and STAMP, respectively, to test the significant differences in specific variables among treatment groups. Pearson correlation analysis was used to reveal the relationship between the activity and abundance of denitrifying bacteria using SPSS (version 20.0). Principal coordinate analysis (PCoA, vegan in R) was conducted to visualize assemblage conditions of the denitrifying bacterial community. Random forest analysis (RANDOMFOREST package in R) was used to evaluate the importance of environmental predictors for denitrifying bacteria. The co-occurrence networks were constructed based on the Molecular Ecological Network Analyses Pipeline (MENA) and visualized with Gephi 0.9.2 to detect the interactions among denitrifying bacteria. Structural equation modeling (SEM) was performed to investigate the direct and indirect effects of parameters on DEAs using Amos 21.

## 3 Results

### 3.1 Physiochemical characteristics of standing water and soil

The physiochemical characteristics in the paddy field differed among distinct straw retention treatment groups (**Table 1**). Inorganic N, especially NH_4_^+^, comprised the largest proportion of N in the standing water. Compared to those with NS treatment, concentrations of NH_4_^+^ and NO_3_^−^ in the standing water significantly decreased, whereas those of DON and DOC increased after straw retention (*P* < 0.05). The conditions in the soil were much different. First, organic N (i.e., SON) took the dominant portion of soil N, followed by NH_4_^+^, NO_3_^−^, and NO_2_^-^. More importantly, compared to those with NS treatment, soil pH and soil NO_3_^−^ and NO_2_^−^ contents decreased, whereas those of NH_4_^+^, SON, SOC, and DOC increased, after the 7-year implementation of straw retention (*P* < 0.05). Furthermore, whether in the standing water or soil, treatment groups with rice straw amendment (RS or WRS) showed more analogous trends. These results revealed that both in the standing water and soil of the paddy field, the NO_3_^−^ value was reduced, and the organic C and N values were enhanced by the practice of straw retention, and particularly by rice straw amendment.

### 3.2 Denitrification enzyme activity and the abundance of *nosZ* denitrifiers

**Figure 1:**
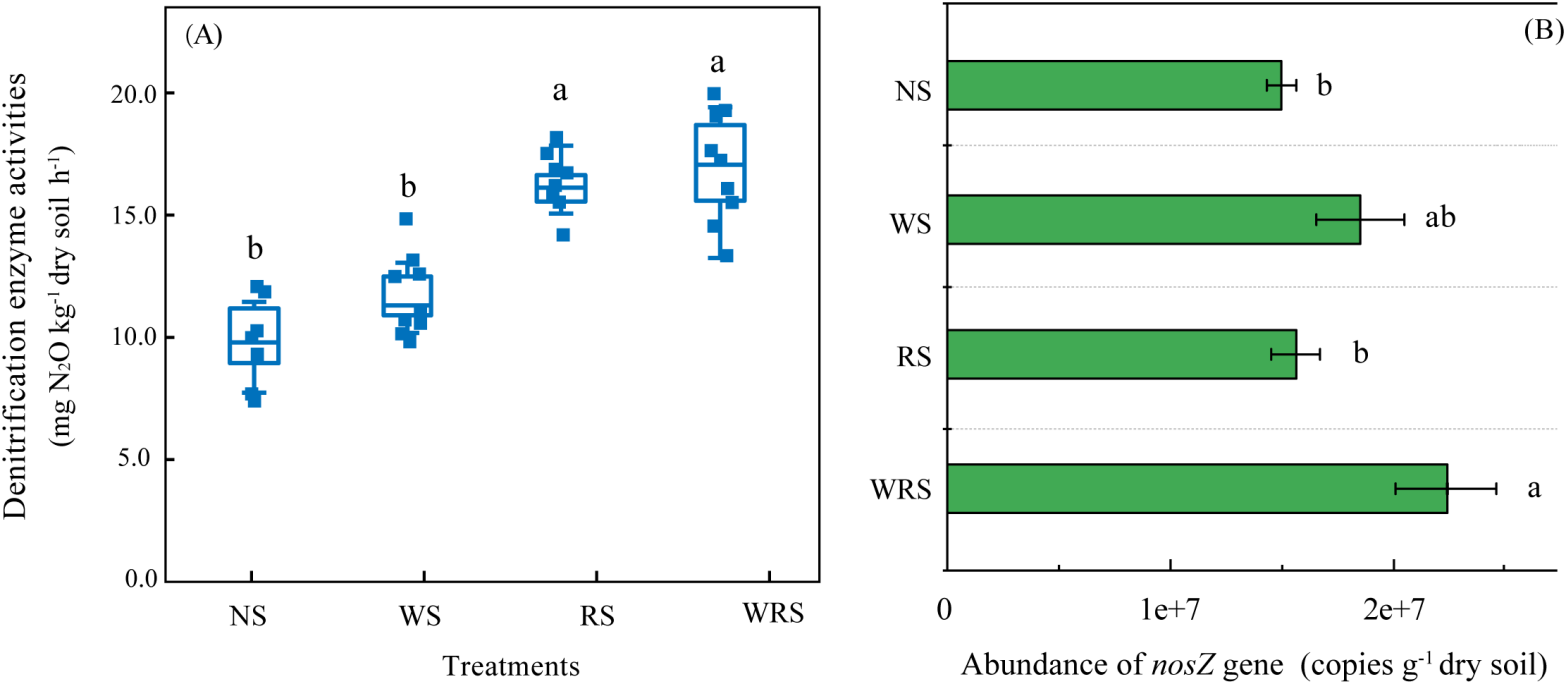
Denitrification enzyme activities **(A)** and the abundance of the *nosZ* gene **(B)** under the four fertilization regimes. Error bars denote the standard errors (n = 3) and are accompanied by different lowercase letters indicating significant differences (*P* < 0.05) among the four treatment groups according to Tukey’s HSD *post-hoc* test. NS, no straw; RS, rice straw only; WS, wheat straw only; WRS, rice straw and wheat straw.

DEA was used to characterize the potential soil denitrification activity. In the current experimental field, soil DEA was observed to vary from 14.48 to 20.55 mg N_2_O-N kg^−1^ h^−1^ (**Figure 1**). Although the DEA value increased as an effect of straw retention, only treatments with rice straw amendment (RS and WRS) resulted in significant differences, of which the increments were estimated to be 41.93% and 45.80%, respectively. These results indicate that the employment of rice straw retention might amplify denitrifying N loss in the rice–wheat rotation system.

Analogous with DEA results, the abundance of the denitrifier-*nosZ* gene, which ranged from 1.52 to 2.27 × 10^7^ copies g^−1^ dry soil, was enhanced with straw retention treatments. However, relative to that with NS, only the WRS treatment group showed significantly higher levels of *nosZ* gene abundance, which also did not correlate with the soil DEA (*P* > 0.05). The investigation into different phylogroups, but not the copy numbers of denitrifiers, thus appears to be particularly important to account for soil DEA changes as affected by crop residues.

### 3.3 Beta-diversity and co-occurrence networks of *nosZ*-denitrifying communities

**Figure 2:**
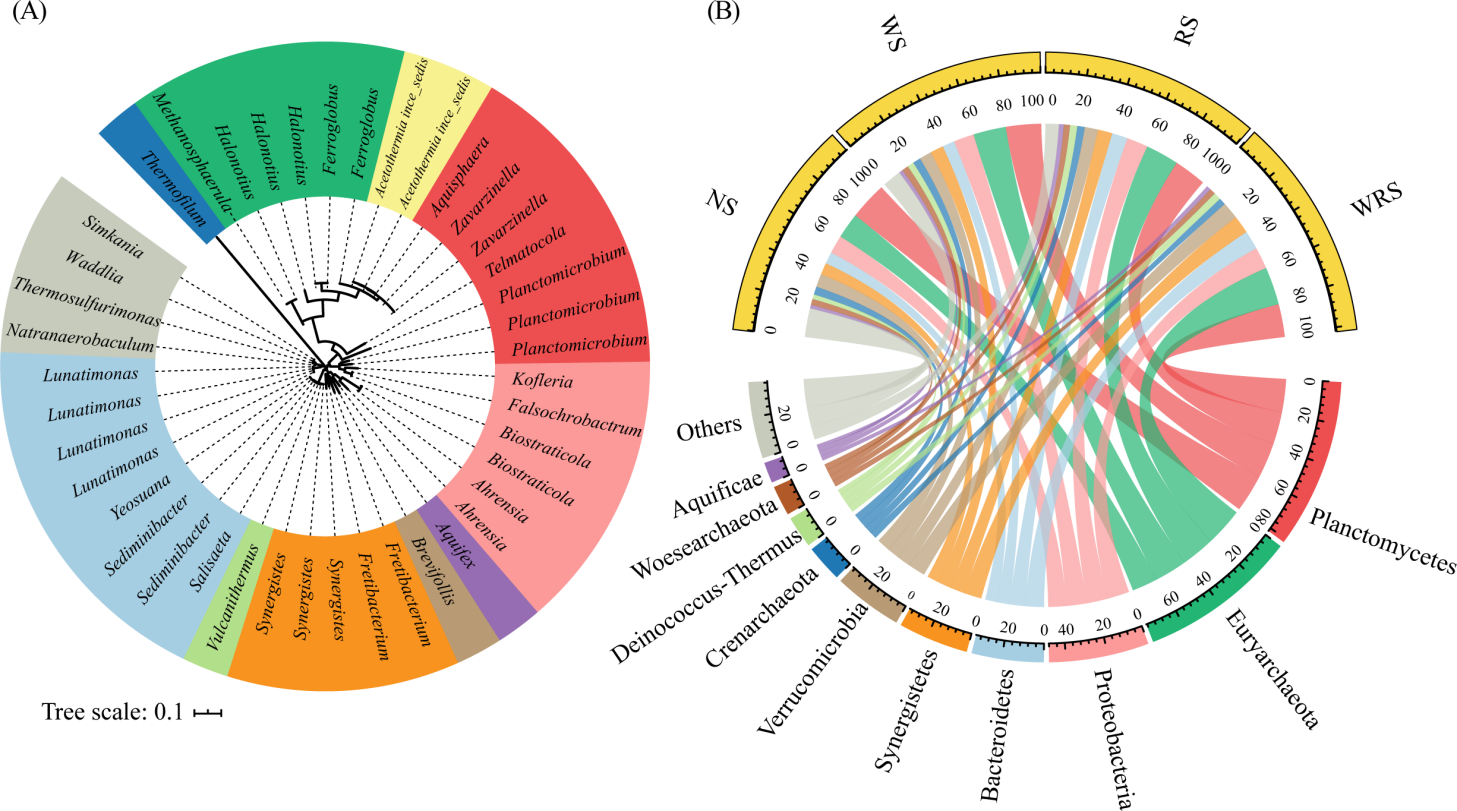
Circular maximum likelihood phylogenetic tree **(A)** and the community compositions **(B)**, presented based on genus and phylum levels, respectively, of the denitrifying bacteria among treatment groups. The tree is based on the *nosZ* gene sequences of the most abundant operational taxonomic units. Genera in **(A)** are color-coded by phylum in **(B)**. NS, no straw; RS, rice straw only; WS, wheat straw only; WRS, rice straw and wheat straw.

**Figure 3:**
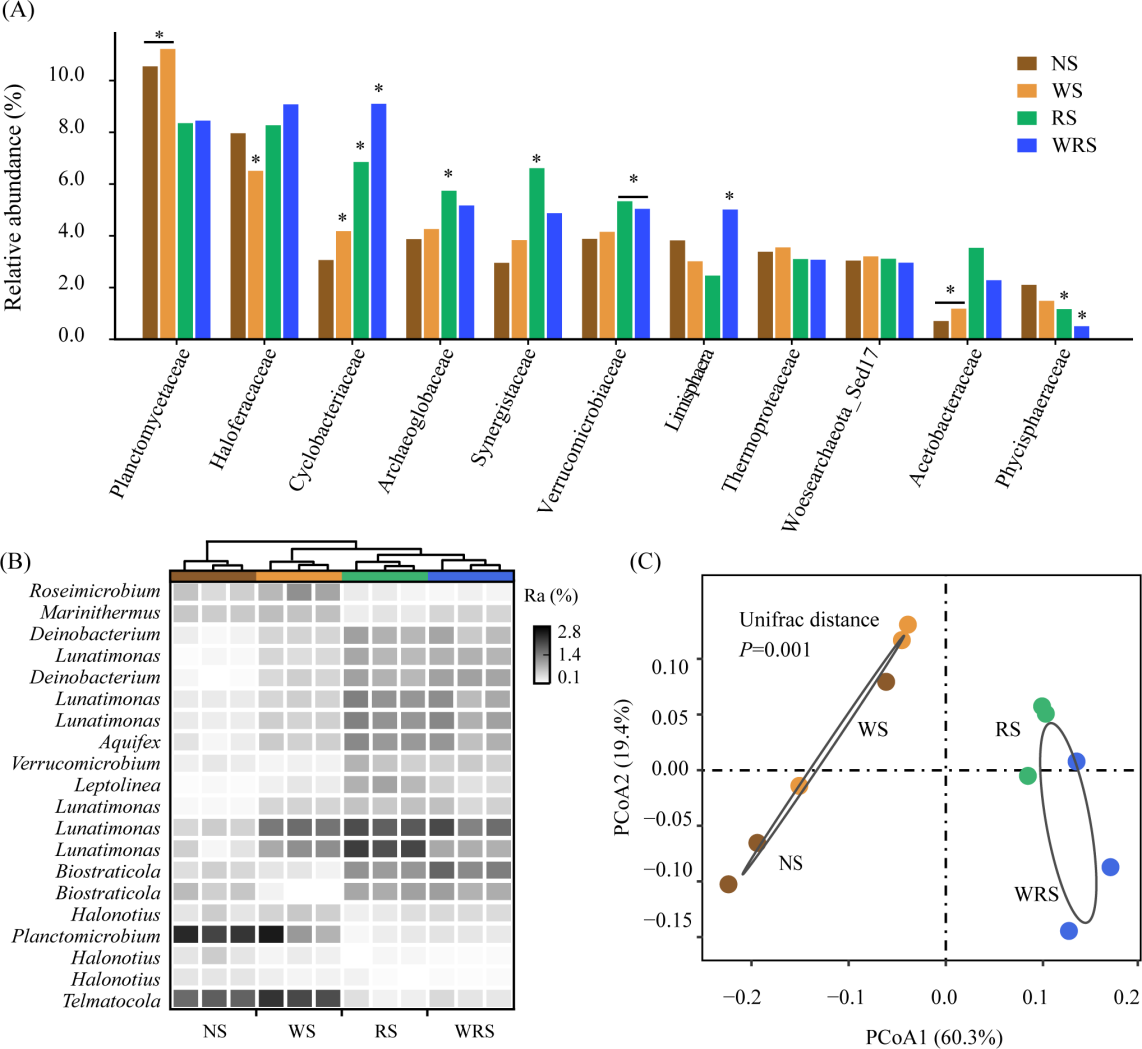
Differences in the community compositions of denitrifying bacteria at family **(A)** and genus **(B)** levels and in the bacterial community structures **(C)** among treatment groups. The statistical differences were calculated using Tukey’s HSD *post-hoc* test (marked with asterisks, *P* < 0.05, n = 3), Kruskal-Wallis H-test (*P* < 0.05, n = 3), and PERMANOVA test (*P* = 0.001, n = 6) in **(A–C)**, respectively. NS, no straw; RS, rice straw only; WS, wheat straw only; WRS, rice straw and wheat straw.

The characteristics of *nosZ*-denitrifying communities showed clear variations in response to different straw retention patterns. In the current study, 2691 clustered OTUs were phylogenetically grouped as 69.9% bacteria and 30.1% archaea (**Figure 2A**), which were predominated by Planctomycetes (19.0%–22.3%), Euryarchaeota (15.1%–22.3%), and Proteobacteria (10.8%–12.7%) at the phylum level (**Figure 2B**). The ANOVA and STAMP results demonstrated that under treatments with rice straw retention (RS and WRS treatments), whether at the family or genera level, *nosZ* denitrifiers affiliated with the phyla Euryarchaeota (Haloferacaceae, Archaeoglobaceae, *Halonotius*) and Bacteroidetes (Cyclobacteriaceae, *Lunatimonas*) increased in relative abundance (*P* < 0.05), whereas those affiliated with the phylum Planctomycetes (Phycisphaeraceae, *Planctomicrobium*, *Telmatocola*) decreased (*P* < 0.05) (**Figures 3A, B**; **Supplementary Table S3**). PCoA and PERMANOVA results further identified the community distribution characteristics of *nosZ* denitrifiers, revealing that RS and WRS groups were significantly differentiated relative to the NS and WS groups (**Figure 3C**).

**Figure 4:**
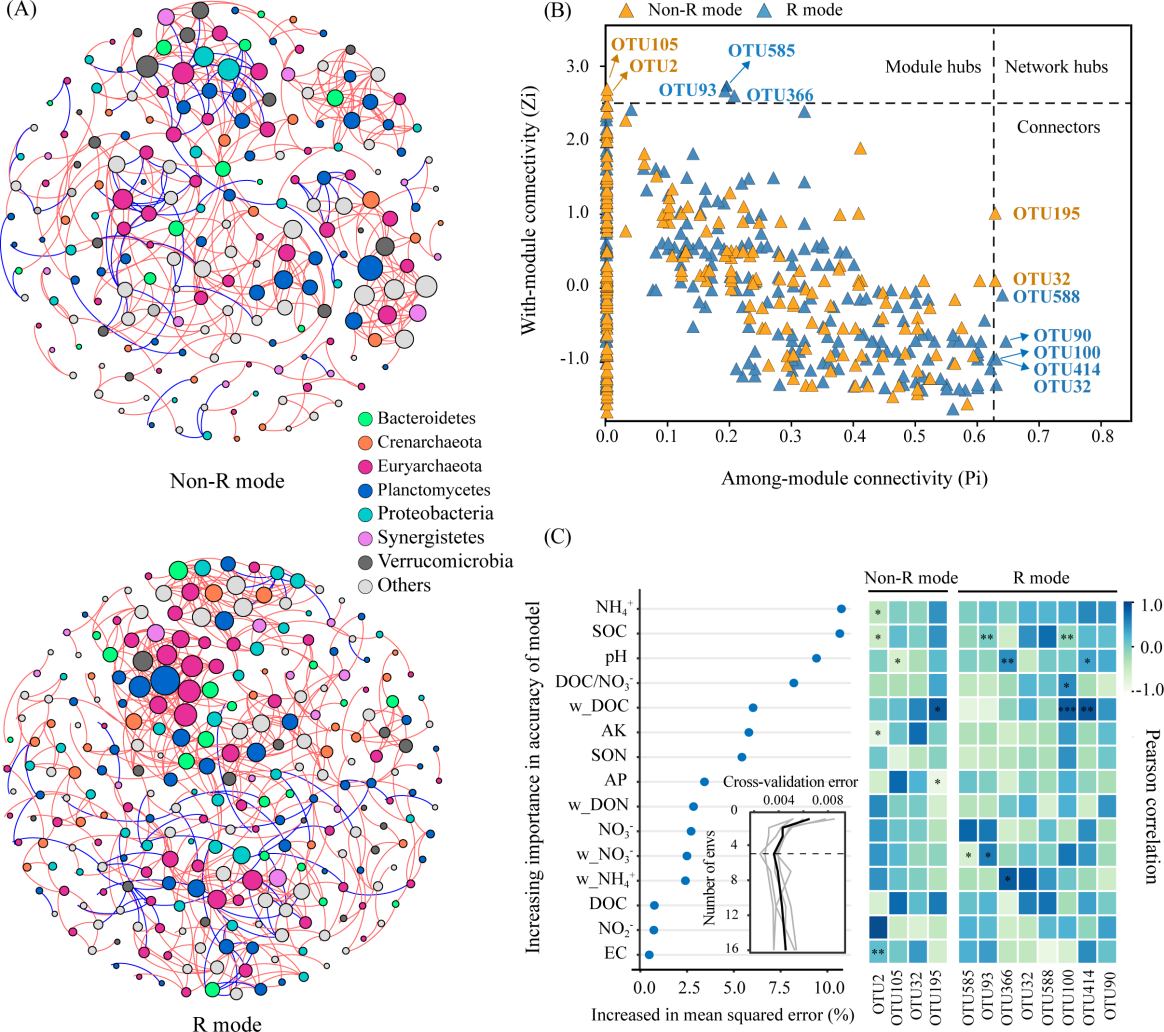
Co-occurrence networks **(A)** and the keystone taxa **(B)** of denitrifying bacterial operational taxonomic units (OTUs) based on their topological roles, and the relationships of keystone taxa with soil environmental predictors calculated based on the random forest model **(C)**. Non-R and R modes integrate treatments without (NS+WS) and with (RS+WRS) rice straw retention, respectively. Nodes in **(A)** are colored based on the phylum level, and their sizes are proportional to the number of degrees; the red and blue edges represent positive and negative correlations between two nodes, respectively, the thicknesses of which are proportional to the value of Spearman’s correlation coefficients. Keystone taxa of co-occurrence networks represented by hubs and connectors in **(B)** are categorized by threshold lines of Zi = 2.5 and Pi = 0.625, respectively. *P*-values < 0.05, < 0.01 and < 0.001 are indicated using *, **, and *** in **(C)**, respectively. NS, no straw; RS, rice straw only; WS, wheat straw only; WRS, rice straw and wheat straw.

These findings suggested that treatments with and without rice straw resulted in pairwise similarity concerning both the community composition and structure of *nosZ* denitrifiers. Correspondingly, R mode and non-rice straw (non-R) mode co-occurrence networks were separately constructed to identify the key phylogroups and interactions among the *nosZ* denitrifiers (**Figure 4**). Generally, non-R and R mode networks consisted of 202 and 262 nodes and 440 and 606 edges, respectively, the latter possessing a higher ratio of positive associations (88.0% > 82.1%), as well as higher average degree and clustering coefficient values, as shown from the topological features (**Figure 4A**; **Table 2**). Correspondingly, the Zi-Pi plot showed four (two module hubs and two connectors) and eight (three module hubs and five connectors) hub nodes (i.e., core OTUs), which are representative of keystone taxa of denitrifiers, within the non-R and R networks, respectively (**Figure 4B**; **Supplementary Table S3**). The keystone taxa include OTUs prominently from the phylum Planctomycetes (OTU2, OTU195) in the W network, whereas OTUs were prominently from the phyla Euryarchaeota (OTU585, OTU93, OTU414) and Bacteroidetes (OTU100, OTU90) in the R+S network. These findings revealed that the *nosZ* denitrifiers in the R mode were more positively interconnected and recruited more and specific keystone taxa, as compared with those in non-R mode.

**Table 2:** Topological properties of co-occurrence networks of denitrifying microbial communities.

Network metrics	non-R mode	R mode
Number of nodes	202	262
Number of edges (Positive/Negative)	440 (82.1%/17.9%)	606 (88.0%/12.0%)
Average degree	4.35	4.63
Clustering coefficient	0.45	0.53
Modularity	0.82	0.81

### 3.4 Associations between denitrification activity and abiotic or biotic environmental factors

**Figure 5:**
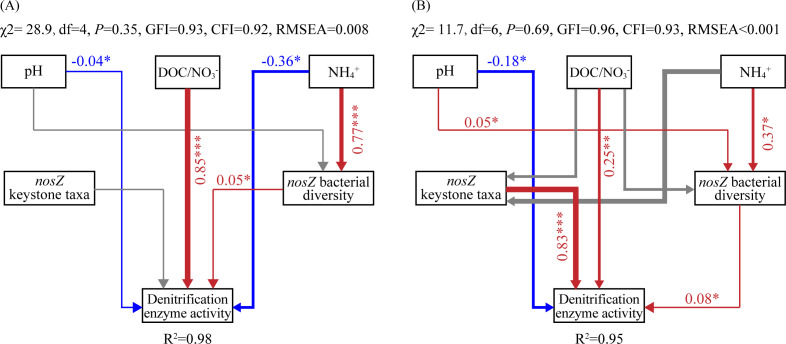
Structural equation models describing the direct effects of physicochemical and bacterial community characteristics of paddy soil on denitrification enzyme activities under non-R **(A)** and R **(B)** modes, respectively.

Random forest analyses and Pearson correlation results demonstrated that the *nosZ* denitrifiers and associated keystone taxa were predominantly adjusted by soil NH_4_^+^, SOC, pH, DOC/NO_3_^−^, and DOC (in order of significance) (**Figure 4C**). On this basis, the direct and indirect effects of environmental factors on soil DEA, under the R and non-R modes, respectively, were assessed with SEM (**Figure 5**; **Table 3**). Generally, more than 90% of soil DEA variance was explained by these two models. Soil DOC/NO_3_^−^ (r_non-R_ = 0.85; r_R_ = 0.03), NH_4_^+^ (r_non-R_ = −0.32; r_R_ = −0.61), and pH (r_non-R_ = −0.04; r_R_ = −0.11) were all key factors in both models, and these directly and indirectly regulated the soil DEA by modulating biotic factors including *nosZ* bacterial diversity (r_non-R_ = 0.05; r_R_ = 0.08) and keystone taxa (r_non-R_ = 0; r_R_ = 0.83). Interestingly, soil DEA was stimulated by a higher level of soil DOC/NO_3_^−^, particularly in the non-R model, whereas it was the *nosZ* keystone taxa (r = 0.83, *P* < 0.001) that generated the largest positive effect on soil DEA exclusively in the R model. This underlines the fact that *nosZ* keystone taxa might play an extremely important role in stimulating soil DEA under rice straw retention conditions.

**Table 3:** Standardized regression weights of direct, indirect, and total effects of the key factors on denitrification enzyme activities.

	pH	DOC/NO_3_^−^	NH_4_^+^	*nosZ* keystone taxa	*nosZ* bacterial diversity
Direct effects	−0.04 | −0.18	0.85 | 0.25	−0.36 | 0	0 | 0.83	0.05 | 0.08
Indirect effects	0 | 0.07	0 | −0.22	0.04 | −0.61	0 | 0	0 | 0
Total effects	−0.04 | −0.11	0.85 | 0.03	−0.32 | −0.61	0 | 0.83	0.05 | 0.08

The numbers to the left and the right of the vertical bar (|) represent the parameters from structural equation modeling of non-R and R modes, respectively.

## 4 Discussion

### 4.1 Variations in soil–standing water properties that respond to straw retention

The availability of N is a key factor affecting crop development and the effect of the environment in an agricultural ecosystem (Ju et al., 2009; Zhang et al., 2015). Both in standing water and soil in the present study, the NO_3_^−^ value was reduced with straw retention treatments (*P* < 0.05) compared to that with NS treatment. This is beneficial for N loss load reductions, particularly for areas at a high risk of runoff, and was consistent with the results of most studies focused on flooded paddy fields (Chen et al., 2023; Bhattacharyya et al., 2012; Leon and Kohyama, 2017). Distinctively, the NH_4_^+^ content in the soil significantly increased after long-term straw retention, particularly for treatments with rice straw amendment (RS and WRS treatments). This was primarily attributed to the characteristics of inorganic N release and microbial immobilization from crop residue decomposition (Kahlon et al., 2013; Xu et al., 2024). More importantly, the available N level, which could be enhanced by stimulation with organic N mineralization following crop residue input, was amplified more with residues having a relatively lower C/N compared to that with residues with a higher C/N (Chen et al., 2021b; Tatti et al., 2017), respectively, corresponding to the rice straw (C/N = 62/1) and wheat straw (C/N = 110/1) in the current study. Nevertheless, the low levels of NO_3_^−^ with straw retention treatments might imply higher activities of NO_3_^−^ microbial reduction processes (such as denitrification, anammox, or dissimilatory nitrate reduction to ammonium) rather than its accumulation (Liu et al., 2020; Wang et al., 2021, 2024; Zhang et al., 2021c). Moreover, it is understandable that organic C and N, either in soil or standing water, were enhanced after long-term straw retention (*P* < 0.05), given the periodic supply of exogenous organic material in conjunction with the amplified microbial activity, which would support the accumulation of organic nutrients (Malhi et al., 2011; Wang et al., 2018; Xu et al., 2024).

### 4.2 Variations in *nosZ* denitrifiers in response to straw retention

The responses of the nitrous oxide reductase gene *nosZ* to fertilization in soil ecosystems have attracted considerable attention (Duan et al., 2018; Li et al., 2022; Philippot et al., 2011; Qin et al., 2021; Tao et al., 2022). In paddy fields, compositions of the dominant *nosZ*-denitrifying community were found to vary widely against divergent backgrounds (Cucu et al., 2017; Wang et al., 2020). In this experimental field, the *nosZ* denitrifiers were predominated by Planctomycetes (19.0%–22.3%), Euryarchaeota (15.1%–22.3%), and Proteobacteria (10.8%–12.7%). Most phylogroups of the latter two phyla derived from the *nosZ* I clade type, which can perform the complete denitrification process (Frostegård et al., 2022; Lin et al., 2023; Zhang et al., 2021a). In contrast, Planctomycetes-affiliated organisms were recently found to belong to the *nosZ* II clade type based on metagenomics-based analysis (Zhuang et al., 2020). The higher positive association ratio (88%) in the R mode network highlights stronger cooperative interactions among *nosZ* denitrifiers under rice straw retention, suggesting a more stable and synergistic community structure that facilitates nitrogen cycling (Tao et al., 2022; Zheng et al., 2019). The increased clustering coefficient further indicates the presence of tightly connected microbial clusters, potentially acting as hotspots for efficient nitrogen transformations. These findings underline the diverse ecological roles of *nosZ* denitrifiers in response to long-term straw retention and the importance of microbial interactions in driving soil nitrogen processes (Hallin et al., 2018; Wei et al., 2015).

Treatments with (R mode) and without (non-R mode) rice straw retention resulted in significant differences, in terms of either the community composition, structure, or keystone taxa of *nosZ* denitrifiers. Generally, the R mode resulted in the recruitment of more groups from Bacteroidetes and Euryarchaeota phyla as dominant bacteria or keystone taxa. For example, the genera *Lunatimonas* (phyla Bacteroidetes) and *Halonotius* (phyla Euryarchaeota), which originate from sea and lake sediments, are typical NO_3_^−^-reducing salinophilic bacteria and archaea (Durán-Viseras et al., 2023; Song et al., 2019; Vavourakis et al., 2016). This indicates that the recruitment of these keystone taxa under rice straw retention likely contributes to the observed improvement in denitrification efficiency, aligning with findings that enriched organic carbon conditions stimulate the activity of keystone denitrifiers (Bano et al., 2024; Chen et al., 2025). In contrast, the predominant taxa *Planctomicrobium* and *Telmatocola*, which belong to the Planctomycetes phylum in non-R mode, include parthenogenetic anaerobic denitrifying bacteria (Cao et al., 2021) and serve as the dominant bacteria in the anammox process (Chen et al., 2021a; Zheng et al., 2019). This further implies that the specific community of *nosZ* denitrifiers in the R mode might thereby increase the competitiveness of the denitrification process with respect to nitrate–nitrogen allotropic reduction by increasing the abundance of uncoupled *Lunatimonas* and *Halonotius* colonies and their positive interaction ratios, which is partially supported by findings from the co-occurrence networks.

### 4.2 Roles of abiotic and biotic environmental factors in soil DEA responses to straw retention

In the present paddy field, treatments with rice straw retention (RS and WRS treatments) rather than wheat straw amendment only (WS treatment) significantly increased the soil DEA (by ~ 41.93–45.80%). This corroborates 3-year observation data showing that the application of rice straw results in significant higher seasonal N_2_O emissions than wheat straw only, under the equivalent input of inorganic fertilizers (Wang et al., 2019). However, neither differences in *nosZ* gene abundance among distinct straw retention modes nor correlation coefficients for DEA and *nosZ* gene abundance reach significance. This represents a prevailing controversy based on many studies (Attard et al., 2011; Kou et al., 2019; Kong et al., 2023; Yin et al., 2015), and perhaps in the future, the relationship between gene expression abundance and reactivity can be determined at the mRNA level.

Without considering the abundance of the *nosZ* gene, the associated microbial community characteristics, as well as the biotic environmental factors, were found to regulate the soil DEA in the experimental paddy field. Specifically, soil DOC and *nosZ* denitrifier diversity produced a positive effect on the soil DEA, and similar phenomena have been found in many ecosystems (Gao et al., 2019; Jiang et al., 2020; Surey et al., 2020; Yeerken et al., 2024). DOC provides electron donors for denitrification processes and improves microbial competition for soil NO_3_^−^-N (Bai et al., 2015; Tao et al., 2022). In contrast, the soil pH, which ranged from 6.63 to 7.10, was partially deviated from the optimum neutral to slightly alkaline range (pH 7–8) (Liu et al., 2010) and had direct negative effects on soil DEA in this study. An interesting finding from this long-term field experiment is that it was rice rather than wheat straw amendment that significantly stimulated soil DEA through the contributions from the keystone taxa of denitrifiers—specifically, a few nitrite-reducing salinophilic bacteria and archaea. This implied that long-term rice straw amendment offers favorable conditions of electron or nitrite competition for these denitrifiers (Wang et al., 2024; Zhang et al., 2021b). The significant role of a few core phylogroups of denitrifiers has been increasingly observed by researchers (Qin et al., 2021; Tao et al., 2022). However, more evidence in terms of microbial cultivation is warranted to directly validate the underlying mechanisms.

## 5 Conclusions

Results of the present study provide evidence of how and why soil denitrification activity vary among different straw retention modes in a paddy field. Under the equivalent input of chemical fertilizers, soil denitrification activity can be significantly improved by long-term rice straw retention (in single or double crop seasons). Key environmental factors including soil DOC/NO^3-^, NH_4_^+^, and pH, along with keystone taxa and the diversity of nosZ denitrifiers, showed distinct variations between rice and wheat straw retention modes. Particularly, specific *nosZ* keystone taxa play a prominent role in driving denitrification activity exclusively under treatment with rice straw retention. By contrast, the practice of wheat straw retention in a single season would be recommended to minimize soil N loss from the denitrification process in a rice–wheat rotation field. Given the complexities of denitrification activity determinants, activity denitrification rates cannot be extrapolated to obtain areal fluxes for a paddy field, and intact core or new *in situ* methods need to be employed and validated in future research.

## Data Availability

The original contributions presented in the study are included in the article/**Supplementary Material**. Further inquiries can be directed to the corresponding author.
