# Supplementary material for: Roles of core nosZ denitrifiers in enhancing denitrification activity under long-term rice straw retention
Source: Front Plant Sci. 2025 Feb 7;16:1541202. doi: 10.3389/fpls.2025.1541202 (PMC11842374; doi:10.3389/fpls.2025.1541202)
Supplement: Supplementary file 1 [file Table1.docx]

**Roles of core *nosZ* denitrifiers in enhancing denitrification activity under long-term rice straw retention**

Shijie Zhang^1,2^, Mengyao Hou^1,2^, Bing Li^1,2^, Panfeng Guan^1,2^, Qing Chi^1,2^, Hao Sun^1,2^, Hangbo Xu^1,2^, Dongjie Cui^1,2^, Yupan Zhu^1,2,*^

^1^ Zhengzhou Research Base, State Key Laboratory of Cotton Bio-breeding and Integrated Utilization, School of Agricultural Sciences, Zhengzhou University, Zhengzhou 450001, China

^2^ Henan Key Laboratory of Ion-Beam Green Agriculture Bioengineering, Zhengzhou University, Zhengzhou 450001, China

Table S1 Field experimental treatments

|  | NS | WS | RS | WRS |
| --- | --- | --- | --- | --- |
| Wheat/rice straw rate (kg dry matter ha^−1^) | 0/0 | 5500/0 | 0/10,000 | 5500/10,000 |
| Chemical N fertilizer rate (kg ha^−1^) | 240/200 | 240/200 | 240/200 | 240/200 |
| Chemical P fertilizer rate (kg ha^−1^) | 15/30 | 15/30 | 15/30 | 15/30 |
| Chemical K fertilizer rate (kg ha^−1^) | 60/30 | 60/30 | 60/30 | 60/30 |

The numbers to the left and the right of the slash (/) represent the application rate in the rice and wheat seasons, respectively. The split N (in the form of urea) and K (in the form of potassium chloride) application ratios were 4:2:4 and 5:0:5, respectively, as basal, tiller fertilizer, and panicle fertilizers. NS, no straw; RS, rice straw only; WS, wheat straw only; WRS, rice straw and wheat straw.

Table S2 Variance analysis of the relative abundance (%) of the top 10 denitrifying bacterial phyla

| Treatment | Planctomy -cetes | Euryarchaeota | Proteobacteria | Bacteroidetes | Synergistetes | Verrucomic -robia | Crenarchaeota | Deinococcus | Woesearch -aeota | Aquificae |
| --- | --- | --- | --- | --- | --- | --- | --- | --- | --- | --- |
| NS | 22.33±1.30a | 15.13±2.20b | 10.80±0.69b | 6.98±0.57c | 6.90±0.57c | 7.80±0.99ab | 4.73±0.38a | 2.77±0.13b | 3.52±0.07a | 2.10±0.52a |
| WS | 20.83±0.49b | 17.30±1.65b | 12.03±0.51ab | 7.20±0.37c | 7.78±0.32bc | 7.25±0.22b | 4.64±0.26a | 3.84±0.65a | 3.79±0.22a | 2.28±0.67a |
| RS | 19.07±0.40c | 19.63±1.17a | 12.53±1.55ab | 9.35±1.09b | 8.49±0.12b | 7.89±0.73ab | 4.4±0.38ab | 4.25±0.72a | 3.65±0.45a | 2.87±0.19a |
| WRS | 19.50±0.20c | 22.37±1.50a | 12.77±0.40a | 11.10±0.62a | 10.56±1.03a | 10.14±2.22a | 4.04±0.08b | 4.35±0.36a | 3.45±0.35a | 2.62±0.05a |

Different lowercase letters in the same column indicate significant differences (*P* < 0.05) according to the Tukey’s HSD post-hoc test. NS, no straw; RS, rice straw only; WS, wheat straw only; WRS, rice straw and wheat straw.

Table S3 Keystone taxa of the denitrifying microbes

| Network | Generalists | Node ID | Node degree | Clustering coefficient | Relative abundance (%) | Affiliation phylum/genus |
| --- | --- | --- | --- | --- | --- | --- |
| non-R mode | Module hubs | OTU2 | 13 | 0.46 | 2.008 | Planctomycetes/*Zavarzinella* |
|  |  | OTU105 | 10 | 0.42 | 0.280 | Euryarchaeota/*Thermogymnomonas* |
|  | Connectors | OTU32 | 4 | 0.17 | 0.475 | Proteobacteria/*Nannocystis* |
|  |  | OTU195 | 5 | 0.20 | 0.097 | Planctomycetes/*Zavarzinella* |
| R mode | Module hubs | OTU585 | 11 | 0.44 | 0.021 | Euryarchaeota/*Ferroglobus* |
|  |  | OTU93 | 12 | 0.47 | 0.229 | Euryarchaeota/*Methanopyrus* |
|  |  | OTU366 | 19 | 0.40 | 0.023 | Planctomycetes/*Zavarzinella* |
|  | Connectors | OTU32 | 5 | 0.31 | 0.282 | Proteobacteria/*Nannocystis* |
|  |  | OTU588 | 5 | 0.32 | 0.765 | Verrucomicrobia/*Verrucomicrobium* |
|  |  | OTU100 | 4 | 0.02 | 1.177 | Bacteroidetes/*Lunatimonas* |
|  |  | OTU414 | 4 | 0.01 | 0.060 | Euryarchaeota/*Halonotius* |
|  |  | OTU90 | 5 | 0.30 | 0.153 | Bacteroidetes/*Phocaeicola* |
